# Alcohol use and sexual risk behaviour among men and women in inner-city Johannesburg, South Africa

**DOI:** 10.1186/s12889-017-4350-4

**Published:** 2017-07-04

**Authors:** Braimoh Bello, Harry Moultrie, Aleefia Somji, Matthew F. Chersich, Charlotte Watts, Sinead Delany-Moretlwe

**Affiliations:** 1 Wits RHI, Faculty of Health Sciences, University of the Witwatersrand, Johannesburg, South Africa; 2 London School of Hygiene and Tropical Medicine, London, UK

**Keywords:** Alcohol use, Sexual-risk behaviours, HIV, South Africa, Heavy episodic drinking, Migrant

## Abstract

**Background:**

Alcohol misuse is a key factor underlying the remarkable vulnerability to HIV infection among men and women in sub-Saharan Africa, especially within urban settings. Its effects, however, vary by type of drinking, population group and are modified by socio-cultural co-factors.

**Methods:**

We interviewed a random sample of 1465 men living in single-sex hostels and 1008 women in adjacent informal settlements in inner-city, Johannesburg, South Africa. Being drunk in the past week was used as an indicator of heavy episodic drinking, and frequency of drinking and number of alcohol units/week used as measures of volume. Associations between dimensions of alcohol use (current drinking, volume of alcohol consumed and heavy episodic drinking patterns) and sexual behaviours were assessed using multivariate logistic regression.

**Results:**

Most participants were internal migrants from KwaZulu Natal province. About half of men were current drinkers, as were 13% of women. Of current male drinkers, 18% drank daily and 23% were drunk in the past week (women: 14% and 29% respectively). Among men, associations between heavy episodic drinking and sexual behaviour were especially pronounced. Compared with non-drinkers, episodic ones were 2.6 fold more likely to have transactional sex (95%CI = 1.7–4.1) and 2.2 fold more likely to have a concurrent partner (95%CI = 1.5–3.2). Alcohol use in men, regardless of measure, was strongly associated with having used physical force to have sex. Overall effects of alcohol on sexual behaviour were larger in women than men, and associations were detected between all alcohol measures in women, and concurrency, transactional sex and having been forced to have sex.

**Conclusions:**

Alcohol use and sexual behaviours are strongly linked among male and female migrant populations in inner-city Johannesburg. More rigorous interventions at both local and macro level are needed to alleviate alcohol harms and mitigate the alcohol-HIV nexus, especially among already vulnerable groups. These should target the specific dimensions of alcohol use that are harmful, assist women who drink to do so more safely and address the linkages between alcohol and sexual violence.

## Background

Alcohol misuse is frequently cited as being one of the key factors underlying the remarkable vulnerability to HIV infection in many parts of sub-Saharan Africa, especially in urban settings [1, 2]. Across the sub-continent, alcohol has been linked with HIV risk via unprotected sex [3], multiple sexual partnerships [4–6], transactional sex [4], and physical and sexual violence [7, 8]. A meta-analysis of 20 African studies showed that both male and female drinkers were about 1.5 fold more likely to be HIV-infected than non-drinkers [9]. This is consistent with global findings [10].

Associations between drinking and sexual behaviour are biologically plausible. Alcohol has psychoactive effects which can result in poor judgement, dampened reasoning skills and a reduction in one’s sense of responsibility [11]. Alcohol alters the brain’s gamma-aminobutyric acid receptors and changes the body’s serotonin levels, resulting in disinhibition – reducing anxiety about the consequences of one’s actions [12]. Assertions about alcohol and sexual behaviour causality are strengthened by studies demonstrating links between drinking contexts and the likelihood of sexual risk behaviours [13]. Meta-analyses have noted that certain drinking contexts, such as bars and other public spaces, portend unprotected sex [14]. Importantly, associations between alcohol and unsafe sex have been demonstrated in event-level data, which assesses alcohol use at the time of specific sexual events [15–17]. Taking all the evidence together, it is clear that associations between alcohol use and unsafe sexual behaviour (and consequent risk for HIV infection) are now well documented [18–21]. Key questions remain unanswered, however. Firstly, a better understanding is needed of the mediating or confounding effects of personality factors such as sensation seeking and impulsivity on the alcohol and sex relationship [22–24]. Secondly, additional evidence is needed across different settings to better elucidate which dimensions of alcohol use influence sexual behaviour, and the extent to which being male or female alters the relative effects of these different dimensions. These dimensions include total volume consumed, drinking patterns such as heavy episodic drinking, and alcohol abuse or dependence [25]. This knowledge will enable alcohol control strategies and specific interventions to reduce the harms of alcohol to be tailored to the particular aspects of alcohol use which are most harmful in each setting.

Levels of alcohol consumption and prevalence of HIV in South Africa rank among the highest in the world [26]. One-third of South Africans report heavy, episodic drinking [27] and rates are even higher in patients attending clinical services[28]. HIV prevalence in urban informal areas in the country is almost 20% [29]. HIV incidence is especially concerning among young migrants in urban and peri-urban settings [29], and alcohol problems pervade these communities, both historically and in more recent times [30–34]. These populations also face challenges in accessing health services and other positive determinants of health including housing and employment [35]. Linkages between alcohol and sexual behaviours in these marginalized groups have, however, not been examined in sufficient detail.

We analysed data from a population-based study among men living in hostels and women inhabiting adjacent informal settlements in inner-city Johannesburg, South Africa, to determine the extent to which alcohol use influences sexual behaviours, and to identify which features of alcohol use have most impact in this population. Data on this population are especially important as the majority are internal migrants (from other parts of South Africa), a key group in the South African HIV epidemic [36, 37].

## Methods

### Study design, population and procedures

The survey, undertaken from January to November 2004, formed part of the initial activities done prior to an intervention project that aimed to improve access to health services for males in single-sex hostels and surrounding informal settlements. The hostels, within Region F, south-eastern Johannesburg, were built in barrack style, and originally created by mining companies to accommodate men from rural areas at minimal cost. They are typically overcrowded, unsafe and lack amenities [38]. After mine closures in the early 1990s, the hostels were occupied largely by unemployed migrants from other parts of South Africa, rather than miners [39]. Shack dwellings often sprung up adjacent to the single-sex hostels and are mostly occupied by women and children.

A community mapping preceded the survey to help understand the layout of the study setting and services in the area. Also, a trained fieldworker spent several nights in a hostel carrying out ethnographic fieldwork in the form of participant observation to provide detailed information on daily life within the hostels, information that was used to formulate the sampling strategy.

Men and women aged 18–55, and resident in the area for at least three months, were eligible for enrolment. A representative sample was obtained through a two-stage sampling process. For men, the first stage entailed random sampling of sleeping units within five hostels (five of the six hostels in the area participated), with the number selected per hostel proportionate to hostel size. Thereafter, in an effort to minimise effects of clustering (for example, where men in each sleeping unit were similar to each other and different from other men), 10% of eligible men in the selected units were sampled. For women, the initial stage was a systematic selection of 10% of all dwellings from a random starting point in the informal settlements. All eligible women in the selected dwelling were then invited to participate. Both informal settlements and hostels were visited several times to maximise participation.

Data were collected through face-to-face interviews in either IsiZulu or Sotho. Informed consent was obtained prior to study procedures.

### Exposure and outcome measures

Alcohol use was assessed in three domains: any alcohol use, volume consumed and heavy episodic drinking patterns [40, 41]. Men or women reporting that they drink alcohol were classified as having any alcohol use. Volume of alcohol consumed was measured by two variables: the frequency of drinking and the total number of standard drinks (10 g/drink) in the past week. The latter was categorised as no drinking, moderate drinking (men: 1–14 drinks, women: 1–7 drinks), problem drinking (men: 15–21 drinks, women: 8–14 drinks) or heavy drinking (men: >21 drinks, women: >14 drinks). The limits for units in the past week are those commonly used to define ‘safe’ alcohol use in non-pregnant adults [42, 43]. Drinking frequency was used as a measure of volume drank as is commonly done [40], whereby cumulative lifetime volume is estimated by a product of drinking frequency and number of units per drinking session. To measure heavy episodic drinking, participants were asked whether they had been drunk in the past week.

Other exposure variables assessed included socio-demographics and health status. Age was categorised as 18–24 years, 25–34 years and ≥35 years. Social cohesion was defined as how close participants felt to others around them (men with other hostels dwellers and women with those in the surrounding informal settlement). This was measured using a Likert scale with five levels, extending from ‘very close’ to ‘not very close’. Perceived safety was also measured using a Likert scale, ranging from ‘very safe’ to ‘very unsafe’. To assess exposure to violence, participants were asked when was the last time they had witnessed violence, with responses classified into whether or not this had occurred in the past three months. Perceived risk for HIV infection was determined by asking participants ‘*What do you think your chances of getting HIV/AIDS are?’*, again measured with a Likert scale, as is commonly done for this indicator [44]. Health status was also measured using a Likert scale from ‘very good’ to ‘poor’.

Sexual risk behaviours, the study outcomes, were assessed through four binary measures. *Condom use at last sex*, regardless of type of partner; *concurrent partnerships (*more than one sexual partner at time of survey); *transactional sex* (men who ever paid for or gave goods for sex, and women who were ever paid or received goods for sex); and *sexual violence,* measured among men as ever having used physical force to have sex, or among women as ever been forced to have sex.

### Statistical analyses

Stata version 13.0 (StataCorp, College Station, TX, USA) was used for analysis. We summarized the data using means and proportions. Differences between categorical variables were identified using chi-square tests and between ordinal categorical variables using the chi-square test for trend. Multivariate logistic regression models were developed to determine whether current drinking, volume consumed and heavy episodic drinking were associated with the four sexual risk behaviour measures defined above. Non-drinkers were used as the reference group in all models. Each model adjusted for age and income, based on a directed acyclic graph for total effect of alcohol consumption on high risk sexual behaviour.

In a post hoc analysis, we described the socio-demographic and other characteristics of the groups of men and women whose drinking patterns were associated with high-risk sexual behaviours. The decision about which group to profile was made on the basis of the multivariate results. In those results, for men, associations between heavy episodic alcohol use (having felt drunk in the past week) and sexual behaviour were more consistent and larger than those for the other indicators of alcohol use. We thus examined the characteristics of men with heavy episodic drinking, aiming to profile those requiring interventions. Among women, we assessed the characteristics of current drinkers, as most female drinkers had high-risk drinking patterns and associations between current drinking and sexual behaviour outcomes were similar to those for the other alcohol variables. Throughout, separate analyses were performed for men and women, as patterns of associations between the exposures and outcomes differed by gender, which is widely acknowledged to be an important modifier of the relationship between alcohol use and sexual behaviour [25, 45].

## Results

### Characteristics of study population

Of males eligible for the survey, 94% enrolled (1465/1559), while only 87% of women did (991/1133). Fifty men and seven women reported never having had vaginal, oral or anal sex and were excluded from analysis. Large differences were observed between men and women for all socio-demographic characteristics compared (all *P* values for comparisons between men and women were <0.001). The mean age of males was 28 years, 86% were single and the large majority were born in KwaZulu Natal (94%; Table 1). Only a third of men had completed secondary school, 5% had never been to school and a further 11% had not finished primary education. Females were on average 29 years old and predominately single (93%). They were mostly from KwaZulu Natal (76%), but 19% hailed from other provinces. Women had even lower education levels than men (12% had never attended school and the same percentage had not completed primary education).

**Table 1:** Sociodemographic characteristics, alcohol use and sexual behaviours of men and women in inner-city Johannesburg

	Men (*N* = 1465)		Women (*N* = 991)		*P*
	Number	%	Number	%	
Age group (years)					
18–24	560	38.5	360	36.3	
25–34	681	46.8	419	42.3	
≥ 35	215	14.8	212	21.4	<0.001
Place of birth					
Gauteng	9	0.6	45	4.6	
KwaZulu Natal	1353	94.3	747	75.5	
Other provinces	72	5.0	187	18.9	
Outside South Africa	1	0.1	10	1.0	<0.001
Completed secondary school					
Yes	473	32.9	224	22.6	<0.001
Married					
Yes	201	14.0	71	7.2	<0.001
Employed					
Yes	631	43.9	236	23.8	<0.001
Income per month (USD)					
0–9	661	46.2	580	58.9	
10–99	225	15.7	246	25.0	
100–199	178	12.4	106	10.8	
≥ 200	368	25.7	53	5.4	<0.001
Currently smoke	666	46.4	63	6.4	<0.001
Current alcohol use^a^	686	47.7	124	12.5	<0.001
Frequency of drinking					
None in past week	1036	75.3	912	93.7	
Once a week	173	12.6	31	3.2	
Several days a week	54	3.9	14	1.4	
Daily	113	8.2	16	1.6	<0.001
Drinking volume in past week^b^					
None in past week	1037	75.9	912	93.9	
Moderate drinking	108	7.9	14	1.4	
Problem drinking	59	4.3	11	1.1	
Heavy drinking	163	11.9	34	3.5	<0.001
Having felt drunk in past week					
Not a current drinker	752	55.1	858	87.4	
No, but are current drinkers	472	34.6	90	9.2	
Yes, felt drunk	141	10.3	34	3.5	<0.001
Ever sex under influence of alcohol	537	37.5	73	7.4	<0.001
Sexual risk behaviors					
Condom use last sex	293	21.4	224	23.0	0.37
Concurrent partners	670	48.1	56	5.7	<0.001
Transactional sex	207	14.7	68	7.0	<0.001
Sexual violence^c^	98	7.0	165	16.8	<0.001

^a^Respondents who say they drink alcohol. ^b^Moderate drinking: men: 1–14 drinks, women: 1–7 drinks; Problem drinking: men 15–21 drinks, women: 8–14 drinks; Heavy drinking: men >21 drinks, women >14 drinks. 1 USD = 6 South Africa Rand. Totals vary due to missing data. ^c^Men who used physical force to have sex and women having been forced to have sex*. P* values compare the distribution in men and women, and are based on chi-square tests

Only 44% of males were employed, and these had a median income of USD 200 per month (IQR = 100–300; 1 USD = 6 South Africa Rand). Even fewer women were employed than men (23%) and their median monthly income was half that of men (USD 100, IQR = 44–163). Men shared their room in the barracks-style hostels with a median eight other men (IQR = 4–13), while women had a median of two companions in their tin shacks (IQR = 1–3).

### Patterns of alcohol use

Among men, 48% reported current alcohol use. As many as 16% of all men had problem/heavy drinking (>14 units/week) and 10% had been drunk in the past week. Of the 686 men who drink, 18% drank alcohol daily and almost a quarter had been drunk in the past week (23%). Men mostly drank beer, a median of 9 units a week (IQR = 7–14).

As with socio-demographic comparisons by gender, all differences between alcohol use in men and in women were large (*P* < 0.001 for all comparisons). Fewer women reported current drinking (13%). However, alcohol use patterns among the 124 women who did drink were especially concerning: 14% drank daily, 29% had been drunk in the past week, and female drinkers consumed a median 7 units a week (IQR = 5–9).

### Sexual risk behaviours and associations with alcohol use

Among the men surveyed, only 21% reported having used a condom at last intercourse. Half the men said they presently had a concurrent sexual partnership (48%), 15% ever had transactional sex and 7% admitted having forced a woman to have sex. Compared with men, a similar proportion of women reported condom use at last sex (23%; *P* = 0.37), but much fewer women reported concurrency or transactional sex (about 6–7% for both measures; *P* < 0.001), and 17% had experienced sexual violence.

In both sexes, none of the alcohol measures were associated with condom use at last sex (Table 2). Consequently, the remaining text in this section pertains only to the other three sexual behaviours assessed. In both men and women, though there was some evidence that alcohol use was associated with sexual behaviour in a dose-dependent manner (for example, heavy episodic drinking and concurrent partners in men, and drinking in the past week and sexual violence among women), on the whole such associations were not evident.

**Table 2:** Multivariate logistic regression models of association between different measures of alcohol use and sexual risk behaviour, by gender

Measures of alcohol use	No condom use at last sex	Concurrent partnership	Transactional sex	Sexual violence^c^
	AOR (95% CI)	AOR (95% CI)	AOR (95% CI)	AOR (95% CI)
MEN (*n* = 1485)				
Current alcohol use^a^				
No	1	1	1	1
Yes	0.92 (0.71–1.19)	**1.60 (1.29–1.98)**	**1.52 (1.13–2.06)**	**2.26 (1.46–3.51)**
Frequency of drinking				
Non-drinker	1	1	1	1
Never in past week	1.05 (0.73–1.50)	**1.56 (1.17–2.08)**	1.13 (0.74–1.72)	**2.37 (1.38–4.05)**
Once a week	0.89 (0.59–1.36)	**1.65 (1.17–2.34)**	**1.70 (1.08–2.69)**	1.72 (0.86–3.43)
> 1 once a week	0.71 (0.48–1.06)	**1.66 (1.17–2.36)**	**1.81 (1.15–2.85)**	**3.35 (1.85–6.06)**
Drinking in past week^b^				
None in past week	1	1	1	1
Moderate drinking	0.79 (0.49–1.27)	**1.60 (1.06–2.43)**	1.36 (0.78–2.37)	1.37 (0.63–2.98)
Problem or heavy use	0.76 (0.53–1.09)	**1.46 (1.08–1.99)**	**1.91 (1.30–2.82)**	**2.10 (1.25–3.51)**
Having felt drunk in past week				
Not a current drinker	1	1	1	1
No, but are current drinkers	0.92 (0.69–1.23)	**1.47 (1.16–1.87)**	1.17 (0.82–1.66)	**2.10 (1.29–3.38)**
Yes, felt drunk	0.82 (0.53–1.28)	**2.18 (1.49–3.21)**	**2.62 (1.67–4.11)**	**4.74 (2.02–6.93)**
WOMEN (*n* = 1008)				
Current alcohol use^a^				
No	1	1	1	1
Yes	0.83 (0.54–1.29)	**4.05 (2.23–7.34)**	**5.94 (3.48–10.1)**	**3.21 (2.11–4.89)**
Frequency of drinking				
Non-drinker	1	1	1	1
Never in past week	0.74 (0.40–1.36)	**4.74 (2.13–10.5)**	**5.33 (2.59–11.0)**	**1.98 (1.04–3.75)**
Once a week	1.81 (0.62–5.30)	2.06 (0.59–7.27)	**6.11 (2.29–16.3)**	**6.09 (2.86–13.0)**
> 1 once a week	-	**4.82 (1.71–13.5)**	**6.18 (2.48–15.4)**	**2.95 (1.34–6.50)**
Drinking volume in past week^b^				
None in past week	1	1	1	1
Moderate drinking	1.04 (0.28–3.83)	1.34 (0.17–10.7)	3.22 (0.68–15.3)	2.64 (0.80–8.68)
Problem or heavy use	0.97 (0.47–2.02)	-	**5.91 (2.78–12.6)**	**4.44 (2.37–8.30)**
Having felt drunk in past week				
Not a current drinker	1	1	1	1
No, but are current drinkers	0.86 (0.51–1.45)	**4.60 (2.35–8.99)**	**5.81 (3.12–10.8)**	**2.84 (1.71–4.71)**
Yes, felt drunk	0.90 (0.40–2.04)	2.19 (0.63–7.56)	**5.43 (2.20–13.4)**	**3.31 (1.59–6.91)**

^a^Respondents who say they drink alcohol. *AOR* adjusted odds ratio, *CI* confidence interval. All models adjusted for age and income based on directed acyclic graph for total effect of alcohol consumption on high-risk sexual behaviour. ^b^Moderate drinking: men: 1–14 drinks, women: 1–7 drinks; Problem or heavy drinking: men ≥15 drinks, women: ≥8 drinks. ^c^Men who used physical force to have sex and women having been forced to have sex. Bold AORs are those where *P* < 0.05. – Sample too small to give an estimate of this adjusted association

Among men, the effect sizes of associations between heavy episodic drinking (having felt drunk in the past week) and sexual behaviour were larger than the effect sizes for the other three measures of drinking (current drinking; frequency of drinking in the past week; and number of alcohol units in the past week). For example, compared with non-drinkers, the odds of having transactional sex were 2.6 fold higher in men with heavy episodic drinking (95% CI = 1.7–4.1), while they were only 1.8 fold in men who drank more than once a week (95% CI = 1.2–2.9). Also of note, for men, associations between alcohol use and having used physical force to have sex were significant with all four alcohol measures, and these effect sizes were larger than that for the other sexual behaviours. Importantly, the odds of perpetrating sexual violence was 4.7 fold higher in men who reported heavy episodic drinking than those who do not drink (95% CI = 2.0–6.9).

With all four alcohol indicators, drinking in females was associated with concurrent partnerships, transactional sex and having been forced to have sex. The point estimates of these associations were high: odds of the sexual behaviours outcomes (other than condom use) were above 5 for 8 of the 24 odd ratios calculated. Odds of transactional sex in the different drinking groups were higher than the odds for the associations between alcohol and the other sexual behaviours assessed in women. Importantly, the odds of the three unsafe sexual behaviours among females who currently drink were 3.2 to 5.9 fold higher than non-drinkers. These figures were similar to those for the other three alcohol variables in most of the associations examined.

### Factors associated with heavy episodic drinking in men and current drinking in women

The proportion of men with heavy episodic drinking rose with each increase in age group, from 6% in men 18–24 years up to 16% in those older than 35 (*P* < 0.001), but episodic drinking was not linked to place of birth or education level (Table 3). Being employed and having an income above 10 USD/month was associated with heavy episodic drinking among men. Levels of heavy episodic drinking were 13% in men who had witnessed violence and 9% in those who had not (*P* = 0.02). Rates of heavy episodic drinking were much higher in men who perceived themselves as having a high risk for acquiring HIV (20%), than those who had a low perceived risk (7%; *P* < 0.001). Health status was not associated with heavy episodic drinking in men, but 16% of those who smoke reported heavy episodic drinking, higher than non-smokers.

**Table 3:** Factors associated with heavy episodic drinking in men and current alcohol use among women in inner-city Johannesburg

	Male heavy episodic drinking (*n* = 141)			Female current drinkers (*n* = 124)		
	Number	Row %	*P*	Number	Row %	*P*
Age group (years)						
18–24	32	6.2	<0.001^a^	33	9.2	0.03^a^
25–34	77	12.1		59	14.1	
≥ 35	32	15.6		32	15.1	
Place of birth						
Gauteng	0	0.0	0.70	10	22.2	<0.001
KwaZulu Natal	131	10.3		73	9.8	
Other provinces	8	12.3		40	21.4	
Outside South Africa	0	0.0		0	0.0	
Completed secondary school						
No	99	10.9	0.36	115	15.0	<0.001
Yes	41	9.3		9	4.0	
Married						
No	119	10.2	0.71	117	12.7	0.48
Yes	21	11.1		7	9.9	
Has children						
No	38	7.6	0.01	19	11.4	0.65
Yes	102	12.0		105	12.7	
Employed						
No	65	8.6	0.03	98	13.0	0.42
Yes	74	12.3		26	11.0	
Income per month (USD)						
0–9	38	6.1		74	12.8	
10–99	33	15.7	<0.001^a^	32	13.0	0.76^a^
100–199	23	13.9		11	10.4	
≥ 200	47	13.2		7	13.2	
Perceived social cohesion						
Very close	79	10.9	0.39^a^	48	12.6	0.96^a^
Close	50	9.0		60	12.6	
Indifferent/not close	11	12.8		16	12.8	
Perceived safety						
Safe or very safe	25	8.1	0.16^a^	29	9.8	0.22^a^
Unsafe	63	10.0		64	14.1	
Very unsafe	51	12.4		31	13.1	
Witnessed violence in past 3 months						
No	90	9.1	0.02	94	11.8	0.09
Yes	50	13.4		30	16.4	
Ever had HIV test						
No	18	13.1	0.23	38	9.4	0.01
Yes	121	9.9		86	14.9	
Perceived risk of acquiring HIV						
No risk at all	44	7.4	<0.001^a^	35	12.9	0.87^a^
Some risk	60	10.5		53	12.2	
High risk	35	19.6		35	12.4	
Current smoker						
No	40	5.5	<0.001	82	8.9	<0.001
Yes	100	16.1		40	63.5	
Health status						
Very good	31	12.4	0.46^a^	19	9.0	0.004^a^
Good or fair	88	9.7		68	11.8	
Poor	22	10.6		37	18.7	
STI symptoms in past 6 months						
No	110	10.1	0.58	44	9.8	0.016
Yes	31	11.2		80	14.8	

1 USD = 6 South Africa Rand. Row percentages are presented, which in men is the proportion of each subgroup who had been drunk in the past week, and in women is the proportion who currently drink in each subgroup. *STI* sexually transmitted infection. ^a^Chi-square test for trend (the remainder are chi-square tests). Totals vary due to missing data

Only 9% of women aged 18–24 were current drinkers, while rates were 14–15% in older age groups (*P* < 0.001). Rates of drinking in women from KwaZulu Natal (10%) were half that of women from Gauteng and other provinces (*P* < 0.001). Only 4% of women who completed secondary school currently drink, compared to 15% of those with less education (*P* < 0.001). In women, current drinking was not associated with perceived risk for HIV. Rates of current drinking rose with a decline in health status (9% in those with very good health, 12% in good or fair health and 19% in those with poor health; *P* = 0.004). Lastly, women with STI symptoms were more likely to drink alcohol than those without such symptoms (15% versus 10%; *P* = 0.016).

## Discussion

This study among men and women who are mostly internal migrants and at high risk for HIV [29], showed that alcohol use is associated with unsafe sexual behaviours in this population, though patterns of associations differ between genders. Being drunk in the past week was the strongest predictor of perpetrating sexual violence in men and of unsafe sexual behaviours more generally. On the whole, alcohol consumption was even more strongly associated with sexual risk behaviour among women than in men. Odds of the three unsafe sexual behaviours among females who currently drink were 3.2 to 5.9 fold higher than non-drinkers, while the corresponding odds were only 1.6 to 2.3 in their male peers. Clearly, this population, with notable levels of socio-economic vulnerability, is marked by very high levels of alcohol and sexual risk behaviours.

As in other studies in South Africa [46, 47], considerably fewer women than men drink, but women who do have especially harmful drinking patterns, which are often characterised by frequent heavy drinking episodes. Moreover, the high unemployment rate and considerably lower income among females may mean that those who drink have to depend on men for alcohol. Older men who had an income were much more likely to drink than younger unemployed men, and thus were probably able to purchase drinks for women and offer them transactional sex. The very strong association between transactional sex and alcohol use supports the assertion that alcohol is linked with sexual and other exchanges between men and women in this population [13, 48, 49]. In one study in Cape Town, South Africa, women with food insecurity had higher sexual risk behaviours than other women, with the associations between food and risky sex being fully mediated through women’s alcohol use [49]. Also, that drinking in females per se, rather than type of drinking as in men, was associated with sexual behaviour, suggests that being present in a drinking venue may be *the* key risk for women, rather than the degree to which they engage with alcohol [48]. In fact, few of the associations showed a dose-dependent relationship between alcohol use in men and sexual behaviour, though such associations have been demonstrated previously [50–52].

The juxtaposition of single-sex male hostels and adjacent to informal settlements populated by impoverished women likely means that informal drinking venues in the areas (also called *shebeens*) play a key role in building social cohesion and cementing connections between these populations. Opportunities for drinking and for sexual encounters often co-exist in particular physical locations and the social dynamics that are generated in these locations [13, 53]. Patrons of drinking venues similar to those in the study site reported entering these places with an expectation of securing casual sex [3]. Sexual partners found at these drinking venues are more likely to have multiple sexual partners and to engage in unprotected sex than partners met elsewhere [54, 55]. These drinking venues are also often physically unsafe [38], perhaps accounting for the high levels of violence witnessed by drinkers in our study.

The linkages between alcohol use among men and using force to have sex, consistent with findings elsewhere [5, 56, 57], warrant discussion. Among males, both heavy alcohol use and sexual conquest may serve as markers of masculinity, which taken to their extremes can extend into sexual violence [58]. Drinking venues that encourage heavy alcohol consumption may thus propagate the social norms that underlie coercion and gender-based imbalances in sexual relationships [48]. Clearly, the links between alcohol and the perpetration of sexual violence, in itself, warrants stronger efforts to control alcohol use in South Africa.

The associations noted in the study between alcohol use and sexual behaviour may be accounted for by sex-related alcohol expectancies [59]. These are the anticipated effects of drinking alcohol, such as increased sexual pleasure and sexual riskiness, which then predict the likelihood of these consequences actually taking place [23, 60, 61]. Alcohol outcome expectancies were not studied in this population, but, potentially, the men and women who believed that alcohol would increase their sexual riskiness, may well have acted out those beliefs, which accounts for their high levels of concurrency and transactional sex. That we did not detect an association between alcohol and condom use might be due to the use of a weak indicator of condom use in the study, but also may reflect the mixed findings noted in other reports of associations between condom use and alcohol, with a few studies actually finding higher condom use among drinkers [51, 62]. Clearly, prospective data based on event–level indicators such as condom use while feeling drunk or after drinking are better suited to elucidating causal pathways between alcohol use and unsafe sex [63, 64].

### Study limitations

The cross-sectional nature of this analysis limits the ability to infer causal associations between alcohol use and sexual risk. For example, women who are victims of sexual violence may drink as a means of coping, rather than having experienced rape as a result of drinking. Also, the settings in which drinking takes place were not assessed; they are key mediators of linkages between alcohol use and sexual behaviour. Further, data on drinking and sexual behaviours were self-reported, and thus subject to recall, social desirability and other biases. However, the consistent nature of the findings across a range of alcohol and sexual behaviour measures suggests that the results are valid, despite these limitations.

## Conclusions

Compared with the general population, the effects of alcohol on sexual behaviours are especially heightened among high-risk populations, such as sex workers and men who have sex with men [63]. This study suggests that these effects also hold true for high-risk male and female migrant groups in inner-city areas. More rigorous interventions, at both local and macro level, are needed to alleviate alcohol harms and to tackle the alcohol-HIV nexus, especially among already vulnerable groups. These should target the specific dimensions of alcohol use that are harmful (such as heavy episodic drinking in men), assist women who drink to do so in a safer manner and address the linkages between alcohol and sexual violence.

## Abbreviations

HIV: Human Immunodeficiency virus+
IQR: Inter-quartile range
USD: US Dollars
